# An enigmatic fourth runt domain gene in the fugu genome: ancestral gene loss versus accelerated evolution

**DOI:** 10.1186/1471-2148-4-43

**Published:** 2004-11-04

**Authors:** Gustavo Glusman, Amardeep Kaur, Leroy Hood, Lee Rowen

**Affiliations:** 1 Institute for Systems Biology, 1441 N 34th St., Seattle, WA 98103, USA

## Abstract

**Background:**

The runt domain transcription factors are key regulators of developmental processes in bilaterians, involved both in cell proliferation and differentiation, and their disruption usually leads to disease. Three runt domain genes have been described in each vertebrate genome (the *RUNX *gene family), but only one in other chordates. Therefore, the common ancestor of vertebrates has been thought to have had a single runt domain gene.

**Results:**

Analysis of the genome draft of the fugu pufferfish (*Takifugu rubripes*) reveals the existence of a fourth runt domain gene, *FrRUNT*, in addition to the orthologs of human *RUNX1*, *RUNX2 *and *RUNX3*. The tiny *FrRUNT *packs six exons and two putative promoters in just 3 kb of genomic sequence. The first exon is located within an intron of *FrSUPT3H*, the ortholog of human *SUPT3H*, and the first exon of *FrSUPT3H *resides within the first intron of *FrRUNT*. The two gene structures are therefore "interlocked". In the human genome, *SUPT3H *is instead interlocked with *RUNX2*. *FrRUNT *has no detectable ortholog in the genomes of mammals, birds or amphibians. We consider alternative explanations for an apparent contradiction between the phylogenetic data and the comparison of the genomic neighborhoods of human and fugu runt domain genes. We hypothesize that an ancient *RUNT *locus was lost in the tetrapod lineage, together with *FrFSTL6*, a member of a novel family of follistatin-like genes.

**Conclusions:**

Our results suggest that the runt domain family may have started expanding in chordates much earlier than previously thought, and exemplify the importance of detailed analysis of whole-genome draft sequence to provide new insights into gene evolution.

## Background

Since the initial description of the *Drosophila *segmentation gene *runt *over a decade ago [1], a small family of runt domain (RD) genes has been described and extensively analyzed in several species. The 130 amino acid long runt domain is very highly conserved and is readily identifiable computationally. RD transcription factors are developmental regulators involved both in cell proliferation and differentiation, and their disruption usually leads to disease [2].

In humans, three different RD genes were identified [3] and named according to various schemes, currently standardized by the human gene symbols *RUNX1*, *RUNX2 *and *RUNX3*. RUNX genes have two promoters (P1 and P2, also called distal and proximal, respectively) [4-7] separated by a long intron; the proximal promoter (P2) is always located within a large CpG island [8]. Extensive alternative splicing giving rise to many isoforms has been described for all RUNX genes [9-11].

Orthologs of all three human *RUNX *genes were identified in mouse. A single RD gene was described in *Xenopus*, presumed to be orthologous to *RUNX1 *[12]. An experimental search for RD genes in fugu showed the existence of a fugu ortholog of human *RUNX2*, and suggested the existence of a single additional RD gene in fugu [13], while a computational search of the fugu genomic sequence revealed three *RUNX *genes [14]. Four RD genes were identified in *Drosophila *[14,15], while a single RD gene exists in *C. elegans *[16], sea urchin and amphioxus [17]. Based on these data, current thought on the evolution of the RD gene family posits that a single RD gene was present in the common ancestor of chordates [17], and this ancestral gene triplicated during early vertebrate evolution, giving rise to the modern *RUNX *gene complement. The proposed mechanism of expansion involved large-scale genomic duplications, identifiable today as large paralogous segments [18]. The proper identification of true orthology relationships is often helpful for inferring gene function and translating knowledge between model organisms and more complex species. Under the current model, simple orthology relationships should be expected among vertebrate *RUNX *genes, but their functional relationship to the ancestral RD gene is unknown. The single known RD gene in *C. elegans *has been shown to be required for the formation of a functional gut; this role has been claimed to be conserved with mouse *Runx3 *[19].

The current availability of genome drafts for several vertebrate species, including *Homo sapiens*, *Mus musculus*, *Rattus norvegicus*, *Canis familiaris*, *Gallus gallus*, *Takifugu rubripes*, *Tetraodon nigroviridis*, *Danio rerio *and *Xenopus tropicalis*, allows us to explore a comprehensive set of vertebrate RD genes and characterize their genomic environments, shedding light on the structure and evolution of this important gene family.

## Results

### The fugu genome has at least four runt domain genes

**Figure 1:**
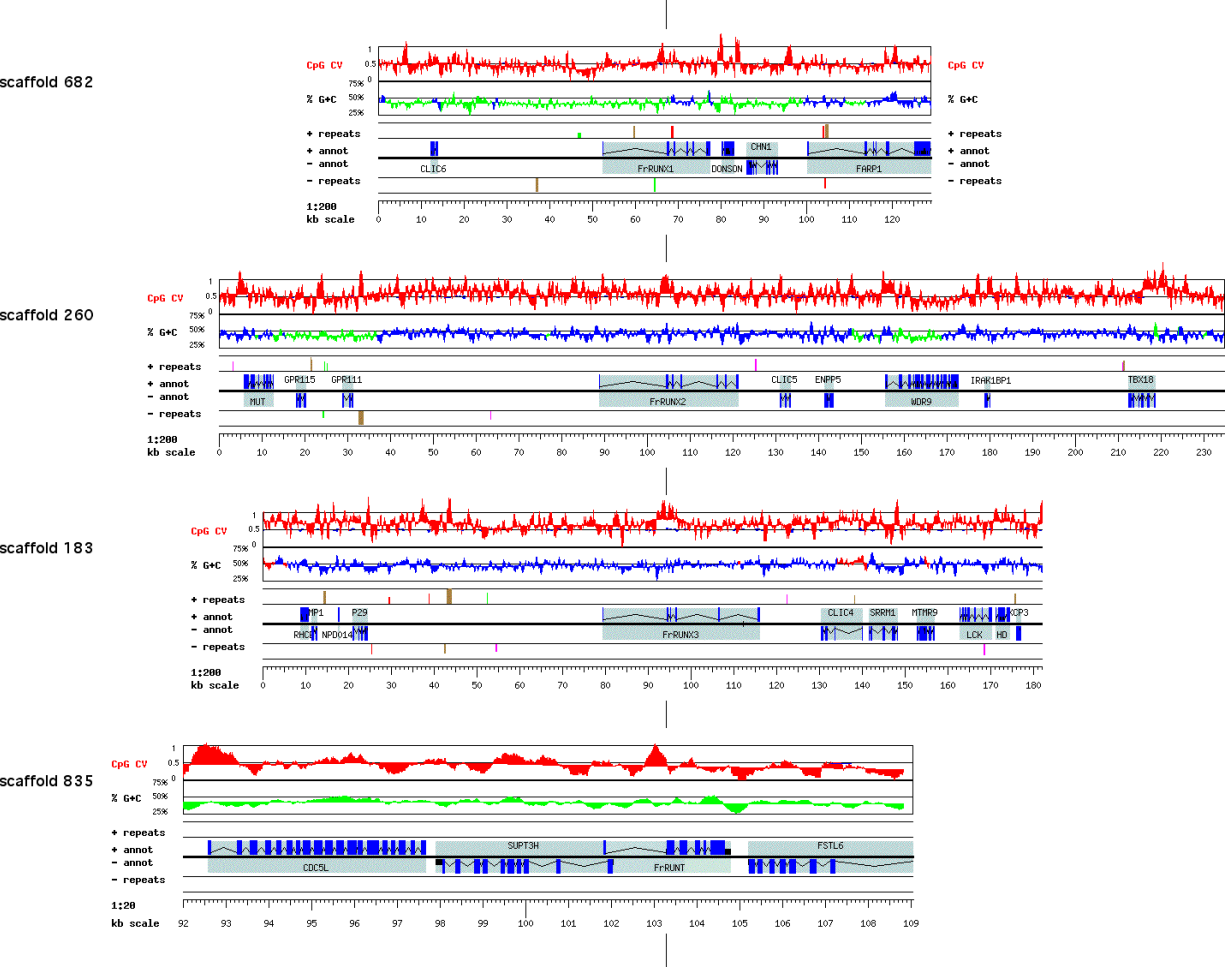
The four fugu scaffolds analyzed and visualized using the GESTALT Workbench. The RD genes are aligned by the position of the proximal promoter. For each scaffold, graphs are shown of the CpG contrast values (observed/expected, typically ~0.5 for the fugu genome), G+C percentage (green – below 43%, blue – between 43% and 50%, red – above 50%), interspersed repeats (SINEs in red, LINEs in green, DNA and LTR elements in brown, other repeats in purple), and gene annotations. Genes and repeats displayed above each black midline are in the forward strand, while those displayed under the midline are in the reverse strand of the sequence. All scaffolds are shown at a resolution of 200 bp/pixel, except for scaffold 835, which includes the tiny *FrRUNT *gene. For clarity, only the last 17 kb of this scaffold are shown at 20 bp/pixel. The much longer scaffold scaffold 183 is also shown truncated at 182 kb; the LCK locus has been studied in detail elsewhere [42].

Our search for RD genes in the fugu draft yielded four distinct genomic scaffolds (Fig. 1 and Table 1), each containing a single, complete RD gene. Each scaffold had one or more sequence gaps, some within the RD genes, others between them and their neighbors. We employed a directed sequencing approach to obtain the additional sequence needed to close the gaps in these four scaffolds and to improve sequence quality.

**Table 1:** The four fugu RD genes and their human orthologs.

Fugu gene				Human ortholog		
Name	Location	Size	%G+C	location	Gene size	%G+C

*FrRUNT*	scaffold_835	3.0	41.2%	N.O.	N.O.	N.O.
*FrRUNX1*	scaffold_682	25.2	42.5%	chr21	262	43.6%
*FrRUNX2*	scaffold_260	32.6	45.7%	chr6	219	39.9%
*FrRUNX3*	scaffold_183	36.8	47.4%	chr1	66	54.8%

Sizes are expressed in kb. N.O.: No ortholog.

We studied the four scaffold sequences using the GESTALT Workbench [20] and constructed hypothetical gene structures for the fugu RD genes by maximizing similarity to known vertebrate RD proteins. Three of the four RD genes found in the fugu genome have clear one-to-one similarity relationships with the three mammalian *RUNX *genes (see phylogenetic analysis below). They have been assumed to be their orthologs [14,17]; we call them *FrRUNX1*, *FrRUNX2 *and *FrRUNX3 *(Fig. 1). Their genomic structures are similar to those of their human counterparts, but their sizes have evolved differently. *RUNX3 *is the smallest of the three human RUNX genes, while in fugu *FrRUNX3 *is the largest (Table 1), and *FrRUNX2 *is significantly larger than *FrRUNX1*. *FrRUNX1 *has acquired an additional intron [17] that is not present in human *RUNX1 *or in any other RD gene. This intron is just 65 bp long, has canonical splice signals, and is in phase 0 with respect to the protein reading frame, at the beginning of the runt domain. An additional intron has been described at the 5' end of the coding region, yielding a short form that would be locally non-homologous to the other RD genes [14]. A detailed comparison of human *RUNX2 *and *FrRUNX2 *has been published [13]. In both human and in fugu, *RUNX3 *has the highest G+C content of the RD genes, while the G+C content of *RUNX2 *differs significantly between the two species (Table 1).

### The fugu RUNT gene

**Figure 2:**
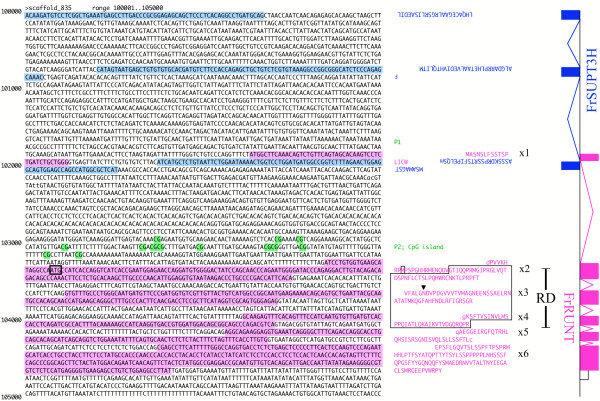
*FrRUNT *sequence detail, showing its compact organization and its interlocking with the *SUPT3H *gene. The two genes are disposed in opposite orientations. The inverted lettering for SUPT3H denotes translation from the reverse strand. The two putative promoters and the extent of the runt domain (RD) are indicated; x1–x6 denote the six exons. The black triangle points at the asparagine residue absent from all previously known runt domain proteins.

In addition to the three *RUNX *genes, the fugu genome has a fourth and more divergent runt-domain gene, that we named *FrRUNT*. *FrRUNT *is an extremely compact gene, spanning just 3 kb of genomic sequence (Fig. 2). Based on sequence analysis only, *FrRUNT *appears to have two promoters, with an intron separating the hypothetical distal promoter (P1) and first exon from the main body of the gene. This intron is usually very long in *RUNX *genes. It is indeed the longest intron observed in *FrRUNT*, but it is nevertheless very short, spanning just 1372 bp. There is a local concentration of CpG dinucleotides 200–300 bp upstream of exon 2 (Figs. 1, 2), suggesting that an incipient CpG island might function as a proximal promoter (P2). The G+C content is not elevated in this area, in similarity to the CpG islands of the fugu *RUNX *genes (Fig. 1). The main body of the gene is split into five exons, separated by much shorter introns (69–190 bp long), all of which have canonical splice signals. The longest predicted FrRUNT product is 294 amino acids long, in contrast with the 496 aa, 463 aa and 421 aa observed for FrRUNX1, FrRUNX2 and FrRUNX3, respectively. The small number of exons in *FrRUNT *leaves little room for alternative splicing by exon skipping, without compromising functionally important domains of the protein. The overall compactness of the gene makes the incorporation of yet undetected exons improbable. Several cryptic splice sites within the exons could enable splicing variants altering exon length.

FrRUNT is exceptional in that the length of the runt domain (131 residues) varies from the universally conserved 130 amino acids, due to the introduction of an asparagine residue after position 47 in the RD (Fig. 2). This appears to be the first report of such a mutation within this highly conserved domain. We also noted that the RD sequence of the tunicate *Oikopleura dioica *(AAS21356 in GenBank) has an insertion at the same position (of two amino acids, a proline and an isoleucine). Comparison to the published structure of this domain [21] shows that this variable region is located in loop L4, opposite the DNA-binding region, i.e. in the location least likely to disrupt the structure of the protein.

A surprising observation is that *FrRUNT *is "interlocked" with *FrSUPT3H *(Fig. 2), the gene orthologous to the human transcription factor *SUPT3H *[22]: The first exon of *FrRUNT *is located within the first intron of *FrSUPT3H*, and vice-versa. In the human genome, though, *SUPT3H *is interlocked with *RUNX2*, as shown in [13]. We discuss the puzzles posed by these differences in genomic organization below.

### Four RD genes in Tetraodon and in zebrafish

**Figure 3:**
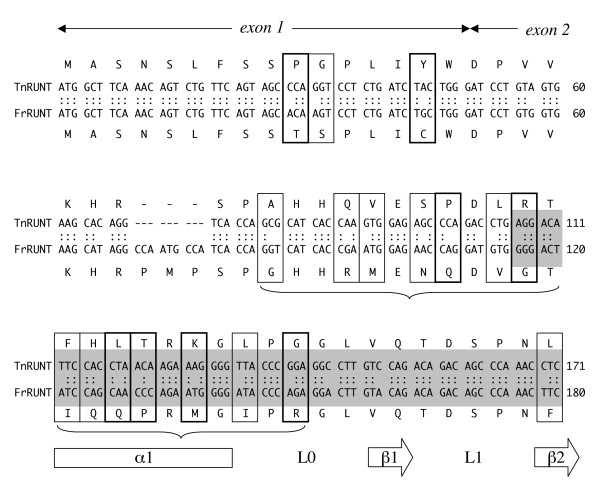
Localized high divergence between TnRUNT and FrRUNT. This alignment between the first 171 bp of the predicted *TnRUNT *coding sequence, with the first 180 bp of *FrRUNT*, shows a cluster of mutations (curly brackets) around the N-terminus of the runt domain (gray background). Non-conservative mutations (negative BLOSUM scores) are surrounded with thick black frames; thin frames enclose conservative mutations, and synonymous mutations are not indicated. The first sixteen codons derive from exon 1, the rest of the alignment from exon 2. The secondary structures indicated (alpha helix, beta strands and loops) are after [21].

A genome draft of another pufferfish species, *Tetraodon nigroviridis*, has been released [23]. A computational search into this draft reveals four RD genes with clear orthologous relationships with the four fugu RD genes. We call them *TnRUNX1*, *TnRUNX2 *and *TnRUNX3*, and *TnRUNT *(Table 2). The RD portion of TnRUNT has been deposited in the EMBL database (accession CAG00330); in this work we report the complete gene structure of *TnRUNT*, which is similar to that of *FrRUNT*. In further similarity with the fugu genomic organization, *TnRUNT *is "interlocked" with the Tetraodon ortholog of *SUPT3H *(not shown). The three *RUNX *gene pairs are conserved between fugu and Tetraodon (Table 2) and display a larger percentage of protein identity than nucleotide identity, indicating a prevalence of conservative substitutions. In contrast, TnRUNT is only 83.3% identical to FrRUNT at the protein level, less than their nucleotide identity. Indeed, a striking series of non-synonymous mutations has created a highly divergent segment (only eight identical amino acids out of twenty-two) including the N-terminus of the runt domain (Fig. 3). The strong and unexpected divergence is not the result of local low sequence quality, as tested by examining the relevant Tetraodon entries from the NCBI Trace Archive, and by resequencing the fugu gene. The differences between TnRUNT and FrRUNT do not modify the Ig-like β-sandwich core of the runt domain [21], which is highly conserved as expected. Therefore, the structural integrity of the runt domain appears not to be compromised. We can only speculate that the extensive variation in its N-terminus may reflect species-specific constraints.

**Table 2:** The four *Tetraodon nigroviridis *RD genes, and their identity levels to their respective fugu orthologs.

Tetraodon gene				Identity	
Name	Location	Size	%G+C	Nucleotide	Protein

*TnRUNT*	SCAF14597	2.5	42.3%	87.5%	83.3%
*TnRUNX1*	SCAF15084	(*) 6.0	44.1%	93.3%	97.0%
*TnRUNX2*	SCAF14590	(*) 31.8	44.4%	96.4%	99.4%
*TnRUNX3*	SCAF15009	(*) 36.6	45.3%	94.6%	98.1%

Sizes are expressed in kb. Asterisks denote minimal sizes due to the presence of gaps within the genes; the gene structure for *TnRUNX1 *is incomplete at its 3' end.

We also searched for RD genes in the genome draft of the zebrafish, *Danio rerio *[24]. Orthologs of RUNX1-3 are present, but no ortholog of *FrRUNT *could be found. This could be due to the incompleteness of the draft sequence. On the other hand, there are two copies of RUNX2, *RUNX2A *and *RUNX2B*, which have been shown to have somewhat different patterns of expression [25]. We next analyze the phylogenetic relationships between all the observed RD genes.

### Phylogenetic distribution of runt domain genes

We performed exhaustive computational searches for RD genes, and in particular for potential orthologs of *FrRUNT*, using the available drafts of the human, chimp, mouse, rat, dog, chicken and frog genomes. In all cases, we identified three clear matches corresponding to orthologs of the three *RUNX *genes. None of these genomes included a potential ortholog of the fugu/Tetraodon *RUNT *gene. In principle, such orthologs might be found in the future within current sequencing gaps or heterochromatic regions, but considering the virtually finished human genome, and the combined coverage of all the genome drafts, we can infer that the *RUNT *gene is absent in mammals, and probably in all tetrapods.

**Figure 4:**
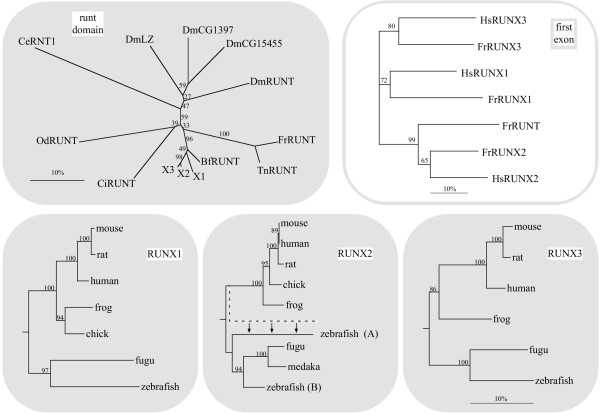
Phylogenetic reconstruction of runt domain evolution. Top left: unrooted tree of RD proteins based on the runt domain; X1, X2 and X3 denote the RUNX clades, BfRUNT, FrRUNT, TnRUNT, CiRUNT, OdRUNT, SpRUNT and CeRNT1 represent RUNT proteins from amphioxus, fugu, Tetraodon, Ciona, *Oikopleura dioica*, sea urchin and *C. elegans*, respectively, while taxa named Dm- represent *D. melanogaster *RD proteins. Top right: unrooted tree of first exons of human and fugu runt domain genes. Bottom: expanded species trees for the three RUNX orthologous groups. For all trees, numbers indicate percent bootstrap support; the horizontal bars indicate 10% divergence along each branch. White and gray backgrounds indicate comparisons at the nucleotide and amino acid level, respectively. The dashed branch in the RUNX2 panel represents the position of zebrafish (A) prior to AsaturA analysis. The arrows indicate the change effected by this correction.

Using representative protein sequences of the RUNX and RUNT genes (see Methods), we reconstructed a molecular tree (Fig. 4, top left) showing the relationship between the three RUNX proteins, FrRUNT and TnRUNT, and the runt proteins of *Ciona intestinalis *and *Branchiostoma floridae *(amphioxus). In this analysis, the FrRUNT protein is nearly equidistant from the three RUNX proteins and amphioxus RUNT (~72% identical, see Table 3). In comparison, the identity level between the RUNX proteins is 90%–98% in the same region (Table 3), and they are 90%–95% identical to amphioxus RUNT. Therefore, while the amphioxus RUNT protein is very closely related to the vertebrate RUNX, the pufferfish RUNT proteins are significantly more divergent.

**Table 3:** Identity matrix.

	FrRUNX2	FrRUNX3	HsRUNX1	HsRUNX2	HsRUNX3	FrRUNT
FrRUNX1	89.7%	91.4%	95.7%	89.7%	89.7%	72.7%
FrRUNX2		95.7%	92.3%	96.6%	96.6%	71.2%
FrRUNX3			94.8%	97.4%	97.4%	72.7%
HsRUNX1				94.0%	94.0%	72.7%
HsRUNX2					98.3%	72.7%
HsRUNX3						72.7%

Percentages of identity between the human and fugu runt domains.

A difficulty has been documented in phylogenetic reconstruction of gene families with anciently duplicated genes [26], in which saturation of frequently-mutating amino acids leads to erroneous "outgroup topologies". In these incorrect topologies, the duplication event appears to be more ancient than supported by the data, which in turn suggest the existence of lineage-specific gene loss events. We tested our phylogenetic reconstruction using the program ASaturA [26], which identifies and suppresses saturated amino acids, thereby correcting the affected tree topology. This analysis did not modify the location of FrRUNT and TnRUNT in our reconstruction, suggesting that mutational saturation is not causing the observed divergence age of the pufferfish *RUNT *genes.

In this protein-level comparison of the conserved runt domains, the pufferfish RUNT proteins appear to be surprisingly ancient, predating the divergence between craniates (including vertebrates) and cephalochordates (including amphioxus). On the other hand, when comparing the nucleotide sequences of the first exon from each human and fugu runt domain gene, we observed that *FrRUNT *is more closely related to *RUNX2 *(Fig. 4, top right), suggesting the possibility of a recombination event between these genes (see discussion below).

We also studied the relationships between the RUNX1, RUNX2 and RUNX3 orthologs in several vertebrates and found the species trees to be largely as expected (Fig. 4, bottom row). One of the two zebrafish RUNX2 protein sequences (RUNX2A) appeared to be slightly more closely related to tetrapod RUNX2 genes than to the other RUNX2 genes in fish species, including zebrafish RUNX2B (dashed branch in Fig. 4, RUNX2 panel). We tested this result using ASaturA [26], and found it to be an artifact of mutational saturation: in the corrected tree (Fig. 4), RUNX2A is more closely related to the other fish RUNX2 genes.

### Comparative genomics of runt domain genes

**Figure 5:**
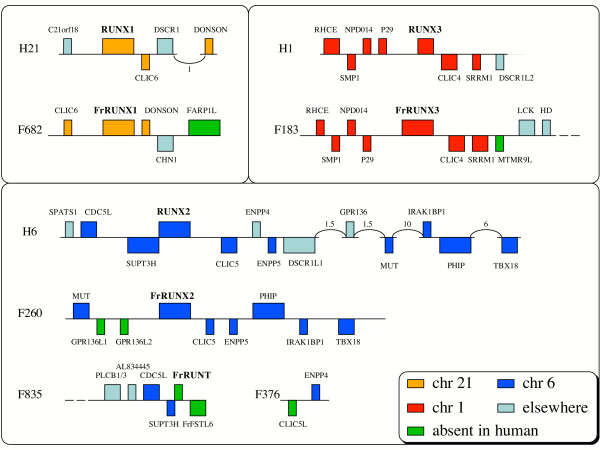
Comparative genomic organization. The genomic neighborhoods of the human and fugu RD genes are compared and contrasted, highlighting synteny conservation (same color between the two species) and gene loss (green features). Feature widths represent rough gene size but are not exactly proportional to gene lengths; likewise intergenic distances are not meant to be precise. Arcs indicate larger genomic distances with one or more intervening genes (not displayed). The numbers associated with the arcs represent the distance in Mb.

Four RD genes have been identified in *Drosophila *[14], more similar to each other than to the vertebrate *RUNX *genes: they represent an independent family expansion in insects. The four *Drosophila *RD genes are all linked on chromosome X. Moreover, three of these genes are clustered within a 150 kb region. There is no linkage of RD genes, however, in the human genome: each gene is on a different chromosome. Their genomic environments usually show some conservation: the three human *RUNX *genes are followed by *CLIC *genes in the complementary strand (Fig. 5), and linked to members of the *DSCR1 *family. The RD genes appear not to be clustered in the fugu genome, though this conclusion is limited by the fragmentary nature of the current genome draft. All four fugu RD genes are flanked by at least one non-RD gene on each side. Fugu *RUNX *genes are followed by *CLIC *genes except for *FrRUNX1*, but a *CLIC *gene is located ~55 kb upstream of *FrRUNX1 *and in the same orientation. This organization could have arisen by an inversion event in the fugu lineage. To ensure a misassembly did not cause this apparent inversion, we performed a 3x shotgun sampling of the BAC clone OML73850, which spans the range 1–97413 of scaffold 682. This quality control step failed to uncover any misassemblies, and confirmed the genomic organization observed in this fugu scaffold. *FrRUNT *does not appear to be linked to any *CLIC *gene, and no DSCR1 family members can be discerned near any of the fugu RD genes.

When studying the wider genomic environments of fugu and human RD genes, we observed significant synteny conservation, in agreement with the observations reported for chromosome X genes [27]. Indeed, when comparing each of the genes neighboring fugu *RUNX *genes to the human genome, we find that their orthologs tend to be located in the corresponding human chromosome, e.g. most of the genes linked to *FrRUNX3 *have orthologs on human chromosome 1, where human *RUNX3 *resides (Fig. 5). Some inversion events can be inferred, e.g. one involving the genes *PHIP *and *IRAK1BP1 *and another involving *MUT*. Gene order and orientation has changed, and intergenic distances have changed drastically, but the overall gene synteny is largely preserved, lending support to the assignments of orthology between the human and fugu *RUNX *gene pairs.

An exception to the conservation of synteny involves the genes *CDC5L *and *SUPT3H*, which in the human genome are found immediately upstream of *RUNX2*, but in fugu are instead located upstream of *FrRUNT*, including the first-exon interlocking with *SUPT3H *mentioned earlier. Evidence points at a larger duplication in fishes, encompassing at least *CDC5L*, *SUPT3H*, *RUNX2*, *CLIC5*, *ENPP4 *and *ENPP5*, followed by differential gene loss. One duplicate copy would have retained *FrRUNX2*, *CLIC5 *and *ENPP5 *(see Fig. 5), while the other copy (currently represented by scaffolds 835 and 376) would have retained *CDC5L*, *SUPT3H*, a second RD gene, a second copy of *CLIC5 *(*CLIC5L*) and *ENPP4*. The presence of remnants of *SUPT3H *upstream of *FrRUNX2 *[13], and of two copies of *RUNX2 *in zebrafish, lends further support to this hypothesis. Thus, comparative genomic analysis of human, fugu and zebrafish suggests that *FrRUNT *may be a derivative form of a duplicated *RUNX2 *gene. However, this contradicts the conclusions from phylogenetic analysis or the protein sequences; we discuss this contradiction below.

### A new family of follistatin-like genes

**Figure 6:**
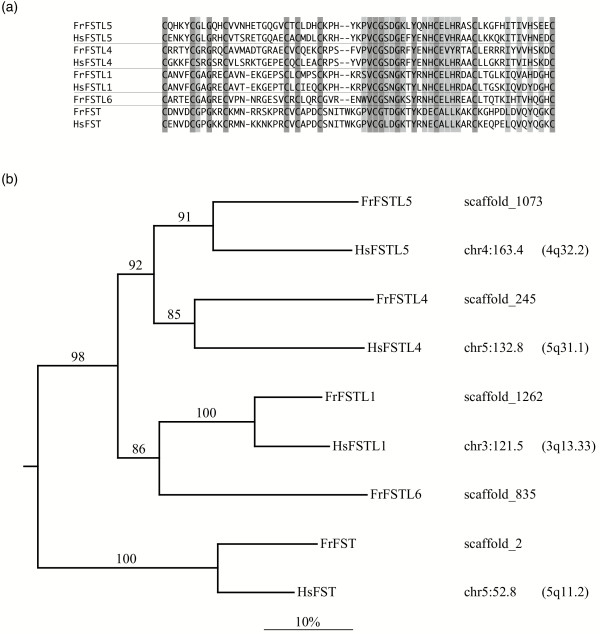
Comparison of selected follistatin-like proteins. FST: follistatin. (a) Multiple alignment of the cysteine-rich domain. Darker shading indicates perfect conservation, lighter shading indicates positions that can be explained assuming one or two mutations. (b) Neighbor-joining tree rooted using human osteonectin/SPARC (NP_003109) as outgroup. Numbers on branches indicate the percent support in 1000 bootstrap replicates. The horizontal bar indicates 10% divergence along each branch. The column on the right indicates the scaffold in which each gene is located (fugu) or its genomic location in Mb coordinates and cytogenetic band (human).

We find several fugu genes for which no human ortholog can be discerned (green features in Fig. 5), among them *FrRUNT*. Immediately downstream to *FrRUNT *we identified a novel gene (*FrFSTL6*) from the follistatin family, most closely related to *FSTL1*. A detailed computational search for additional sequences of this family identified two novel, large human follistatin-like genes, which we called *FSTL4 *and *FSTL5*. We then found clear orthologs for both in the fugu genome (*FrFSTL4 *and *FrFSTL5*, respectively). The genes in this family share a Kazal-type cysteine-rich domain (Fig. 6a) and a calcium-binding EF-hand domain (not shown).

Having characterized the complete gene family in both human and fugu, we performed a phylogenetic reconstruction based on the conserved Kazal-type domain (Fig. 6b). *FrFSTL6 *appears to have no ortholog in the human genome, nor could we identify a potential ortholog in the mouse and frog genomes. This suggests that *FrFSTL6 *was also lost in the tetrapod lineage. It is reasonable to hypothesize that the neighboring *FrRUNT *and *FrFSTL6 *genes were lost in a single deletion event.

## Discussion

We have taken advantage of the availability of genomic drafts for several vertebrate species, including the finished human genome, to identify the orthologs of all currently known runt-domain (RD) genes, as well as a novel member of this small gene family. Both pufferfish species (*Takifugu rubripes *and *Tetraodon nigroviridis*) have four RD genes; since these genomes are only available as draft assemblies, additional RD genes might be found when the finished genomic sequences are made available.

The function of the novel *FrRUNT*/*TnRUNT *gene is currently unknown. Based on the phylogenetic analysis, this novel gene appears to represent an ancestral form of the RD family in vertebrates, subsequently lost in the tetrapod lineage. It is therefore surprising that its gene structure, and not that of *RUNX2 *as in humans, is interlocked with the *SUPT3H *gene. Based on the comparative genomics analysis alone, one could hypothesize that *FrRUNT *is simply a derivative form of *RUNX2*, i.e. the ortholog of zebrafish *RUNX2A*. In this case, though, one would expect FrRUNT to be more similar to FrRUNX2 than it is to either FrRUNX1 or FrRUNX3, but in terms of amino acid sequence similarity, it appears to be equidistant from the three *RUNX *genes. This discrepancy might be explained by invoking accelerated evolution of the pufferfish RUNT genes, perhaps as a lineage-specific adaptation. Typically, nucleotide sequences diverge much faster than amino acid sequences, and the first exons of RD genes are significantly less conserved than the runt domain itself, on which we based our phylogenetic analysis. Therefore, we find it hard to sustain that, while the first exon of *FrRUNT *maintains its nucleotide similarity to the first exon of *RUNX2*, the amino acid sequence of the (normally highly conserved) runt domain itself has diverged at such an accelerated pace. Furthermore, we found this not to be an artifact of mutational saturation [26]. A similar situation is observed for the neighboring *FSTL6 *gene: parsimony considerations could lead one to assume that *FSTL6 *is a fish-specific duplicate of *FSTL1*, though contradicting the phylogenetic reconstruction of the evolution of this gene family (Fig. 6). This situation could again be explained by assuming accelerated evolution of *FSTL6*, but we consider this to be a remarkable coincidence.

**Figure 7:**
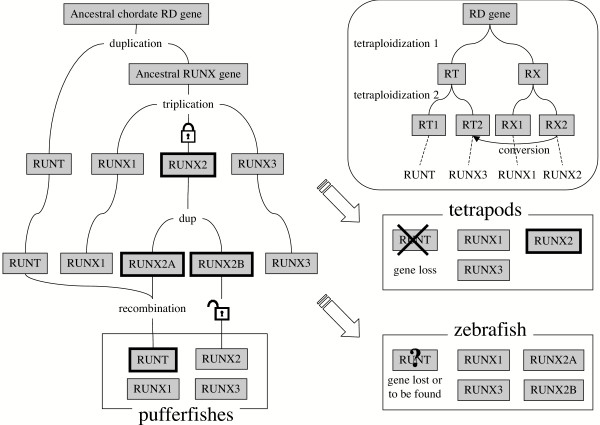
Hypothesis for the evolution of vertebrate RD genes. Features with thick edges represent RD genes interlocked with *SUPT3H *family members. The closed padlock icon represents the interlocking event between the *RUNX2 *and *SUPT3H*, and the open padlock represents the degradation of a copy of *SUPT3H*, releasing *RUNX2 *from interlocking. Not enough information is yet available to establish which of the zebrafish *RUNX2 *genes is interlocked with a *SUPT3H *gene. "dup": duplication. Top right inset: Hypothesis of how the ancestral RD duplication could map to the first vertebrate tetraploidization event. RT and RX represent the ancestral forms of RUNT and RUNX genes.

**Figure 8:**
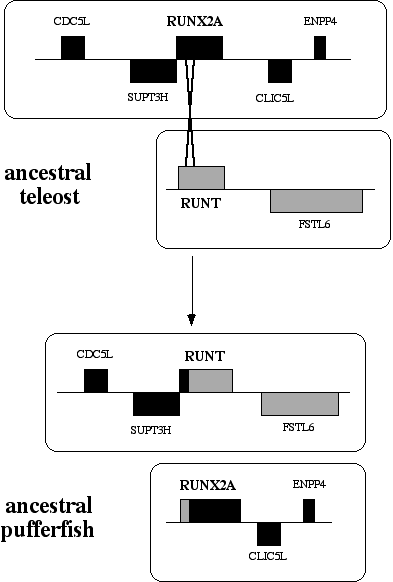
The hypothesized recombination event between the ancestral *RUNX2A *and *RUNT *genes. After the recombination, the *RUNT *gene is linked to *SUPT3H *and has a RUNX2-like first exon. The derivative *RUNX2A *gene has not yet been observed in pufferfishes, and may have been lost in evolution.

The conundrum is whether *FrRUNT *is an ancestral form, or is derived from *RUNX2*. Both hypotheses contradict part of the available data. We propose here a third hypothesis, in the form of an evolutionary history (see Fig. 7): An ancestral RD gene duplicated in chordates, after divergence from sea urchin, which has a single RD gene [17]. One of the two resulting RD genes became the *RUNX *family founder, which expanded by triplication, and one of the three *RUNX *genes (namely *RUNX2*) became interlocked with *SUPT3H*. After the teleost/tetrapod divergence, a regional duplication in teleosts created a second copy of *RUNX2 *and its neighboring genes. In the tetrapod lineage, the ancestral *RUNT *gene was lost, in conjunction with *FSTL6*. In the pufferfish lineage, the *RUNT *gene replaced most of *RUNX2A*, perhaps by recombination (Fig. 8). This is supported by the clear similarity between the first exons of *FrRUNT *and *RUNX2*. The copy of *SUPT3H *interlocked with *RUNX2B*, apparently superfluous, is being lost by gradual degradation, and only small fragments of it remain [13]. This scenario is compatible with all the data observed. While it posits a small number of additional evolutionary events, it does not involve highly improbable events like the accelerated evolution of a normally highly conserved protein structural domain. Interestingly, the first duplication event could correspond to the first round of vertebrate genome tetraploidization [28]. A second round of tetraploidization in the ancestral vertebrate could have produced a set of four paralogous runt domain genes, and a hypothetical gene conversion event may have led to the current complement of three RUNX genes (Fig. 7 inset). Conversion between paralogous copies of genes derived from tetraploidization events has been demonstrated [29].

Under the proposed scenario, none of the three extant *RUNX *genes in mammals represents the ancestral vertebrate RD form. Rather, these derivative genes coexisted with an additional *RUNT *gene and still do so in teleost genomes. The single known RD gene in amphioxus is more similar to the vertebrate *RUNX *genes than it is to *FrRUNT*. It is possible, therefore, that cephalochordates (including amphioxus) have a second RD gene, short and divergent enough to escape experimental detection by DNA hybridization [17]. Why was one of the ancestral RD genes lost in the tetrapod lineage? The three *RUNX *genes bind to the same DNA motif and modify the expression of target genes through recruitment of transcriptional modulators, which are also shared [30]; functional differences between the three *RUNX *genes are attained by way of tightly regulated spatiotemporal expression patterns. We hypothesize that the ancestral *RUNT *gene became inessential to amniotes by functional reprogramming of the remaining three *RUNX *genes. Its loss would therefore represent an example of evolution by reduction in complexity. In pufferfishes, the hypothesized recombination event would have placed the *RUNT *gene under the regulatory control of the former *RUNX2A *promoter. The viability of such a sudden regulatory change would in turn suggest a significant level of functional redundancy among the RD genes.

## Conclusions

We identified a fourth runt domain gene in the fugu genome, which appears to represent either a pufferfish-specific, fast-evolving derivative of *RUNX2*, or a direct descendant of the ancestral chordate *RUNT *gene. We find the latter hypothesis more reasonable. This novel gene evolved in parallel with the vertebrate *RUNX *genes, and while it has been preserved in pufferfishes, it appears to have been lost entirely in tetrapods. This suggests that the ancestral vertebrate was more complex than previously suspected.

By studying a very limited set of fugu genomic regions, namely the scaffolds related to RD genes, we have identified seven apparently functional fugu genes that are absent from the human genome (Fig. 5), and were probably lost early in tetrapod history. In the process of identifying relevant homologs for one of these genes (*FrFSTL6*), we have identified a new family of follistatin-like genes in the human genome. Phylogenetic analysis of the RD protein sequences led to results that contradict those derived from comparative genomics, but we showed that the two could be reconciled into a coherent evolutionary model. These results underscore the importance of obtaining complete genomic sequences of strongly divergent vertebrates, and the value to be derived by performing detailed and integrated analyses of their gene complements.

## Methods

### Search for RD genes

We used the human RUNX1, RUNX2 and RUNX3 proteins (SwissProt entries Q01196, Q13950 and Q13761, respectively) as queries in a TFASTY [31] search into the *Takifugu rubripes *"assembly3" genome draft [32] released after publication of the fugu genome [33]. These data have been provided freely by the Fugu Genome Consortium for use in this publication only. This search resulted in the unambiguous identification of four complete RD genes in scaffolds 183, 260, 682 and 835. Scaffold 25789 is nearly identical with range 115299–115845 of scaffold 183, partially overlapping the last exon of *FrRUNX3*. No further evidence was found for an additional *RUNX3 *gene: we conclude that scaffold 25789 is an assembly artifact. We similarly searched the genome drafts for *Tetraodon nigroviridis *produced by the Whitehead Institute and the Genoscope [23], and *Danio rerio *(Zv1/06 assembly, which was produced by the Zebrafish Sequencing Group at the Sanger Institute [24]. We analyzed, visualized and annotated all resulting genomic sequences using the GESTALT Workbench [20,34], and produced a detailed gene model for each RD gene. Lacking cDNA or EST data, we reconstructed the putative gene structures by maximizing similarity to known RD proteins. Genomic sequence data have been submitted to GenBank with accessions AY739093-AY739096; the predicted sequences for fugu RD proteins have accessions AAU14190-AAU14193.

In a second round of analysis, we used the newly identified RD genes as queries for renewed TFASTY searches of the genome drafts of human (July 03), mouse (February 03), Xenopus [35] (December 03 assembly) and *Ciona intestinalis *[36]. We also used BLAT to search into the updated "freezes" of human (May 04), chimp (November 03), mouse (May 04), rat (June 03), dog (July 04), and chicken (Feb 04).

### Sequence finishing

Large insert clones spanning the gaps in scaffolds from the version 3 assembly were identified by BLAST searches against the BAC/cosmid database in the v.3.0 JGI website [37]. Cosmids (cloned in Lawrist4) were grown in LB media with kanamycin at 37 ° C for 14 hrs, and DNA was prepared on the Autogen 740 DNA Isolation system in accordance with the manufacturer's instructions. BACs (cloned in pBeloBAC 11) were similarly prepared by growing in media with chloramphenicol. Primers were designed in both directions, across all gaps. Oligonucleotide-directed sequencing from clones and Polymerase Chain Reaction (PCR) methods were used to fill the gaps. PCR amplification was performed on spanning BAC/cosmid or genomic DNA of *Takifugu rubripes*, generously provided by Dr. Greg Elgar. PCR products were purified with sephacryl (Amersham Pharmacia) and sequenced directly using Applied Biosystems Big dye terminator kit reagents. Whenever necessary, additional pairs of primers were designed for oligonucleotide-directed sequencing to close gaps. Shotgun sequencing data was obtained from the BAC clone OML73850 (b193C08) for part of scaffold 682. OML73850 was fragmented by sonication, end-repaired and electrophoresed to select insert size of 2–5 kb. Insert was ligated into pUC18 vector, transformed and plasmid DNA was made using Eppendorf – 5 Prime PERFECTprep robot and sequenced from both ends. Assembly was carried out using Phrap [38]. Analysis of the resulting sequence shows that OML73850 spans the first 97413 bp of scaffold 682, and links it to scaffold 4260. The additional sequence data generated in-house were combined with the consensus sequences of the scaffolds produced by the JGI WGS assembly v.3 for the purpose of producing a contiguous sequence for each scaffold.

### Phylogenetic reconstruction

The sequences were aligned using ClustalW [39]. Phylogenetic trees were built using the neighbor-joining algorithm [40] and tested with 1000 rounds of bootstrapping. Graphics were produced with TreeView [41]. Since full-length protein sequences cannot be reliably aligned for extremely divergent RD genes, we used only the runt domain to reconstruct the relationship between the pufferfish RUNT, human RUNX1, RUNX2 and RUNX3 (NP_00175, NP_033950 and NP_004341, respectively), ciona (*C. intestinalis*) RUNT, *Oikopleura dioica *RUNT (AAS21356), amphioxus (*B. floridae*) RUNT (AY146617), sea urchin *S. purpuratus *RUNT (NP_999779) and the four *Drosophila melanogaster *RD sequences (NP_523424, NP_511099, NP_572693 and NP_608398). The tree was rooted using the *C. elegans *RUN protein (AB027412) as outgroup. We further excluded the first thirteen amino acids of the runt domain, to avoid the topological distortion expected in this region from the highly divergent pufferfish RUNT sequences. For the separate phylogenetic trees of the three RUNX genes, we used the complete protein sequences, with gap opening and extension penalties of 5 and 0.1, respectively. ASaturA analyses were performed using PAM250, Kimura's correction and a cutoff value of 9, with 1000 rounds of bootstrap. The first exons of human and fugu runt domain genes were compared at the nucleotide level. For each exon, we selected the range from 30 nucleotides upstream of the ATG codon, to 15 downstream of the splicing donor site, i.e. 103 nucleotides for each *RUNX *gene and 94 nucleotides for *FrRUNT*.

## Authors' contributions

GG conceived of the study, performed the bioinformatics analyses and prepared the manuscript. AK generated the sequence data required for finishing. LH, the laboratory director, provided guidance and contributed to the preparation of the manuscript. LR supervised the Institute for Systems Biology's contributions to the Pufferfish Finishing Consortium and contributed to the preparation of the manuscript. All authors read and approved the final manuscript.
